# Antioxidant Activity in Extracts of 27 Indigenous Taiwanese Vegetables

**DOI:** 10.3390/nu6052115

**Published:** 2014-05-23

**Authors:** Pi-Yu Chao, Su-Yi Lin, Kuan-Hung Lin, Yu-Fen Liu, Ju-Ing Hsu, Chi-Ming Yang, Jun-You Lai

**Affiliations:** 1 Department of Nutrition and Health Sciences, Chinese Culture University, Taipei 11114, Taiwan; E-Mail: crfnhs@dep.pccu.edu.tw (J.-I.H.); 2 Department of Applied Science of Living, Chinese Culture University, Taipei 11114, Taiwan; E-Mails: sui.karen@msa.hinet.net (S.-Y.L.); crrgel@dep.pccu.edu.tw (Y.-F.L.); 3 Graduate Institute of Biotechnology, Chinese Culture University, Taipei 11114, Taiwan; E-Mails: rlin52714@yahoo.com.tw (K.-H.L.); theresapi@yahoo.com.tw (J.-Y.L.); 4 Research Center for Biodiversity, Academia Sinica, Nankang, Taipei 11106, Taiwan; E-Mail: cmyang@gate.sinica.edu.tw

**Keywords:** indigenous plants, antioxidant activity, flavonols

## Abstract

The objectives of this study were to identify the antioxidants and antioxidant axtivity in 27 of Taiwan’s indigenous vegetables. *Lycium chinense* (Lc), *Lactuca indica* (Li), and *Perilla ocymoides* (Po) contained abundant quercetin (Que), while *Artemisia lactiflora* (Al) and *Gynura bicolor* (Gb) were rich in morin and kaempferol, respectively. Additionally, *Nymphoides cristata* (Nc) and *Sechium edule* (Se)-yellow had significantly higher levels of myricetin (Myr) than other tested samples. Cyanidin (Cyan) and malvidin (Mal) were abundant in Gb, *Abelmoschus esculentus* Moench (Abe), Po, *Anisogonium* *esculentum* (Retz.) Presl (Ane), *Ipomoea batatas* (Ib)-purple, and *Hemerocallis fulva* (Hf)-bright orange. Relatively high levels of Trolox equivalent antioxidant capacity (TEAC), oxygen radical absorption capacity (ORAC), and 1,1-diphenyl-2-picryl-hydrazyl (DPPH) radical scavenger were generated from extracts of *Toona sinensis* (Ts) and Po. Significant and positive correlations between antioxidant activity and polyphenols, anthocyanidins, Que, Myr, and morin were observed, indicating that these phytochemicals were some of the main components responsible for the antioxidant activity of tested plants. The much higher antioxidant activity of Po, Ts, and Ib (purple leaf) may be related to their higher Cyan, Que, and polyphenol content.

## 1. Introduction

Vegetables are rich in flavonoids and other pigments. Increased vegetable consumption has been widely promoted because of the health benefits of many non-nutrient phytochemicals associated with health maintenance and prevention of chronic diseases and cancers. Numerous groups of phytochemicals in vegetables such as β-carotene, ascorbate, α-tocopherol, flavonol, and polyphenols are recognized for their antioxidant activity [1]. Among the various vegetables, some phenolics are ubiquitous compounds found in all plants as secondary metabolites [2]. Some endemic species are of particular interest because they may be used for the production of raw materials or preparations containing phytochemicals with significant antioxidant capabilities and health benefits. Some of Taiwan’s indigenous vegetables, such as purple leafy sweet potato, perilla, Chinese knotweed, *Gracilaria tenuistipitata*, lettuce, pea sprouts, and gynura, are favored by Taiwanese for self-health promotion that presumably includes anti-oxidation [3,4,5,6,7], cholesterol reduction [8], inhibition of NO formation [9], and even blood pressure reduction [10]. Previously, we demonstrated that purple-leaved sweet potato (*Ipomoea batatas* Lamark) has free radical scavenging activity and is high in polyphenols and flavonoids [11] and purple-leaved perilla (*Perilla frutescens* Britton) contains relatively high levels of anthocyanidins [12]. In addition, methanolic extracts of the welwet plant, purple-leaved sweet potato, heartleaf houttuynia (*Houttuynia cordata* Thumb.), and purple-leaved perilla are much better at prolonging the lag phase of low density lipoprotein (LDL) oxidation, inhibition of conjugated diene formation and inhibition of malondialdehyde formation [12].

As our understanding of the role of free radicals in human diseases has deepened, antioxidants have attracted broader interest because of their role in inhibiting free radical reactions and their help in protecting the human body against damage by reactive oxygen species (ROS) [13]. Previously, we demonstrated with human aortic endothelial cells (HAECs) and human aortic smooth muscle cells (HASMCs) that purple sweet potato leaf extract (PSPLE), quercetin, cyanidin and chlorophyll-related compounds (CRCs) influence the expression of pro-inflammatory cytokines, adhesion molecules, and ROS-sensitive nuclear transcription factors [14,15]. Since some of Taiwan’s indigenous vegetables contained high contents of quercetin and anthocyanidins [12,16], it is still not clear whether quercetin and cyanidin are also functionally related to other inflammatory cytokine-induced single pathways and gene expressions in HASMCs. Therefore, the objective of this study was to evaluate the antioxidant substances (mainly flavonols and anthocyanidins) and antioxidant activity of different extracts from 27 selected indigenous Taiwanese vegetables. These plants may have excellent potential as sources of natural antioxidants. Our current study explores the relationship between the composition of flavonols and anthocyanidins and antioxidant activity. 

## 2. Experimental Section

### 2.1. Chemicals

Methanol, ethanol, acetone, hydrochloric acid, formic acid, sodium chloride (NaCl), di-sodium hydrogen phosphate, potassium dihydrogen phosphate, Trolox, and butylated hydroxyltoluene were purchased from Merck (Darmstadt, Germany). Myricetin, morin, quercetin, kaempferol, delphinidin, cyanidin, pelargonidin and malvidin were obtained from ROTH (Rheinzabern, Denmark). Other reagents such as gallic acid, 2,2-azino-*bis*-(3-ethylbenzothiazoline-6-sulfonicacid) (ABTS), peroxidase, H_2_O_2_, sodium carbonate (Na_2_CO_3_), Folin & Ciocalteu’s phenol reagent, aluminum chloride, sodium acetate, 2,2-diphenyl-1-picrylhydrazyl (DPPH), methylated β-cyclodextrin (RMCD), 2,2-azobis (2-methylpropionamidine) dihydrochloride (AAPH) and fluorescein (FL) were purchased from Sigma Chemicals (St Louis, MO, USA).

### 2.2. Plant Materials and Preparation of Plant Extracts

The following 27 indigenous vegetables were used: *Gynura bicolor* DC. (Gb), *Sanicula lamelligera* Hance (Sl), *Anredera cordifolia* (Ten.) Steenis (Ac), *Lactuca indica* var. *idivisa* Mak. Hara (Li), *Artemisia lactiflora* Wall. (Al), *Solanum nigrum* (Sn), *Abelmoschus esculentus* Moench. (Abe), *Ocimum basilicum* (Ob), *Perilla ocymoides* L. var. *crispa* Benth. f. purpurea Makino (Po), *Anisogonium* *esculentum* (Retz.) Presl (Ane), *Toona sinensis* (Juss.) M. Roem. (Ts), *Ipomoea batatas*-purple (Ib-purple), *Ipomoea batatas*-green (Ib-green), *Asplenium antiquum* Makino (Aa), *Lycium chinense* Mill. (Lc), *Saccharum officinarum* (So), *Potamogeton pectinatu* (Pp), *Basella alba* (Ba), *Hemerocallis fulva-*green (Hf-green), *Hemerocallis fulva*-bright orange (Hf bright orange), *Amaranthus mangostanus*-green (Am-green), *Amaranthus mangostanus*-red (Am-red), *Nymphoides cristata* (Roxb.) O. Kuntze (Nc), *Sechium edule* (Jacq.) Swartz-yellow (Se-yellow), *Sechium edule* (Jacq.) Swartz-green (Se-green), *Momordica charantia* var. abbreviata Ser. (Mc), and *Brassica campestris* L. ssp. chinensis (Bc). All plants were purchased from local food markets of Neihu and Nankang districts of Taipei City, Taiwan.

The edible leaves of each vegetable were divided into eight individual batches, lyophilized by freeze-drying in a Freeze Dryer (FD-5060, Panchum Scientific Corp., Taipei, Taiwan), ground to powder, and stored at −80 °C until use. Extracts were prepared according to Harnly *et al.* [17]. Briefly, 5 g of powder was refluxed at 75 °C for 5 h in 50 mL of acidified methanol (1.2 N HCl) with 0.4 g/L BHT and filtered through Whatman grade No. 1 qualitative filter paper. Extracts were then concentrated in a rotary vacuum evaporator (R205, Buchi, Flawil, Switzerland), re-suspended in acidic methanol to 6 mL, and stored at −20 °C until antioxidant testing with high-performance liquid chromatography (HPLC).

### 2.3. Determination of Polyphenols, Total Flavonols, Total Flavonoids, and Anthocyanidins

Polyphenol content was determined according to the method of Taga *et al.* [18]. Briefly, standard gallic acid and an aliquot of the acidic methanolic extract were diluted with acidified methanol solution containing 1% HCl. Two mL of 2% Na_2_CO_3 _were mixed into each sample of 100 μL and allowed to equilibrate for 2 min before adding 50% Folin-Ciocalteu reagent. Absorbance at 750 nm was measured at room temperature using the Varioskan Flash Multimode Reader (Thermo Scientific, Rockford, IL, USA). The standard curve for gallic acid was used to calculate polyphenol levels. Total phenolics were expressed as the mg gallic acid equivalent (GAE)/g of dry weight. The standard curve equation was *y* = 0.4995*x* − 0.011, where *R*^2^ = 0.9944. Total flavonols in plant extracts were estimated based on the method of Kumaran and Joel Karunakaran [19]. Briefly, 80% of ethanol containing 1% HCl of solvent was used to extract the lyophilized vegetable samples in a shaker for 2 h at room temperature, then centrifuged at 1430× *g* for 15 min at 4 °C, and repeated extraction three times. Two mL of 2% AlCl_3_ ethanol and 3.0 mL (50 g/L) sodium acetate were added to 2.0 mL acidic ethanolic extracts. Absorption at 440 nm was read after 2.5 h at 25 °C. Sample extracts were evaluated at a final concentration of 0.1 mg/mL. Total flavonols were calculated as a quercetin equivalent (mg/g) and expressed as the mg quercetin equivalent (QUE)/g dry weight using the standard curve equation *y* = 4.5549*x* + 0.0099 (*R*^2^ = 0.9989). Total flavonoids were determined using the method of Ordonez *et al.* [20]. A half-milliliter of 2% AlCl_3_-ethanol solution was added to 0.5 mL of the acidic ethanolic extract and absorbance measured at 420 nm after standing for 1 h at room temperature. Extract samples were evaluated at a final concentration of 0.1 mg/mL. Total flavonoids were expressed as mg QUE/g dry weight, and the standard curve equation was *y* = 1.8823*x* + 0.0313 (*R*^2^ = 0.9971). The anthocyanidin content was determined by the method of Mancinelli *et al.* [21]. Briefly, a solution of 99% methanol and 1% HCl was used to extract lyophilized plant samples in a shaker for 1 h at room temperature followed by centrifuging at 4 °C under 1430× *g* for 15 min and repeated three times. Supernatants were then measured at absorbances of 657 nm and 530 nm. Anthocyanidin (unit/g) = (A_530_ − 0.33 A_657_) × mL of extraction/g DW.

### 2.4. Flavonols and Anthocyanidins Analysis by HPLC

One mL of acid hydrolysate methanolic extract was passed through a 0.45 μm filter prior to injecting 20 μL of it into an HPLC. Samples were analyzed with a Hitachi D-2000 containing a Photodiode Array Detector (L-2455) (Hitachi High-Tech, Tokyo, Japan) and an ODS column (250 × 4.6 mm, 5 μm; YMC, ODS-A, YMC, Kyoto, Japan). The mobile phase consisted of acetonitrile-water (30%:70%; *v*/*v*) containing 1% phosphate acid. The eluent was 100% in 30 min at a flow rate of 1.00 mL/min. The spectrum was recorded at 365 nm for flavonols [22]. Two running solvents with different volumes used and delivered at a rate of 1 mL/min: solvent A was 90% water and 10% formic acid, and solvent B was 22.5% acetonitrile, 22.5% methanol, 40% water, and 10% formic acid. The gradient program used was as follows: 0 min, 20% of B solvent; 0.1~30 min, 28% of B solvent; 30.1~40 min, 70% of B solvent; 40.1~45 min, 100% of B solvent [23]. The spectrum was recorded at 520 nm for anthocyanidins determination. Flavonols (myricetin, morin, quercetin, and kaempferol) and anthocyanidins (delphinidin, cyanidin, pelargonidin, and malvidin) were used as standards.

### 2.5. DPPH Radical, TEAC, and ORAC Assays

The scavenging activity of the DPPH radical in extracted samples was determined according to the method of Shimada *et al.* [24]. Briefly, an aliquot of 1 mL of a methanolic extract with series dilution was added to 1 mL of 0.8 mM DPPH freshly prepared in methanol, mixed well, and left to stand for 30 min before measuring absorbance at 517 nm. The scavenging effect percentage was calculated as [1 − (OD 517 nm)/(control OD 517 nm)] × 100. The IC_50_ of the scavenging effect percentage was then calculated.

The total antioxidant capacity of hydrophilic and lipophilic antioxidants was determined using the horseradish peroxidase catalyzed oxidation of 2,2-azino-*bis*-(3-ethylbenzothiazoline-6-sulfonic acid) (ABTS) [25]. The reaction mixture contained 0.5 mL of 1000 µM ABTS (in ddH_2_O) and 3.5 mL of 100 μM H_2_O_2_ (in 0.1 M PBS). The reaction was started by the addition of 0.5 mL of 44 U/mL peroxidase (in 0.1 M PBS). After 1 h, 0.05 mL of sample extract (ethanolic or methanolic extracts for lipophilic and hydrophilic TEAC quantification, respectively) was added to the mixture. Absorbance was measured at 730 nm after 10 min. Trolox was used as a standard and the total antioxidant capacity of the sample extract measured. The TEAC value was expressed as μmol Trolox per gram dry weight.

For the lipophilic antioxidant assay, the dried hexane extract was dissolved in 250 μL of acetone and then diluted with 750 μL 7% RMCD solution (50% acetone to 50% water, *v*/*v*) [26]. Any further dilution was with the 7% RMCD solution. The 7% RMCD solution was used as a blank and to dissolve the Trolox standards for the lipophilic assay. For sample extract lipophilic analysis, 20 μL of this solution was added to each well in a 96 well microplate. Two hundred μL of fluorescein solution was added to the microplate reader followed by 75 μL of 63.4 mM AAPH (17.2 mg/mL and 9.4 μmol/well), and readings were initiated immediately. Any further dilution of the hydrophilic fraction (acetone/water/acetic acid extract) during the ORAC assay was made with the phosphate buffer. A 20-μL portion of the diluted sample extract was added to each well in a 96-well microplate. The fluorescein solution and AAPH were added in the same manner as in the lipophilic assay, except that 75 μL of 31.7 mM AAPH was added to the assay mixture [27].

### 2.6. Statistical Analysis

Data are expressed as the mean ± standard deviation, and statistical significance was analyzed using a one-way ANOVA followed by Tukey’s Range Test at the 0.05 significance level. Pearson’s linear correlation was also determined. The means of three biological replicates are reported.

## 3. Results

### 3.1. Antioxidant Composition

Table 1 documents the content of various antioxidant substances in the leaves of tested plants. Leaves of Ts plants contained the highest levels of polyphenols (81.86 ± 1.48 mg gallic acid/g DW), while Po levels were 40.80 ± 0.92 mg gallic acid/g DW. Flavonoids, flavonols, and anthocyanidins were abundant in Po at levels of 82.42 ± 7.34 mg quercetin equivalent/g DW, 28.43 ± 1.23 mg quercetin equivalent/g DW, and 471.48 ± 36.40 unit/g DW, respectively. In general, purple-leaved plants such as Po, Ib (purple leaf), and Gb were rich in polyphenols, flavonoids, flavonols, and anthocyanidins. However, anthocyanidins were not detected in Sl, Ac, Li, Al, Sn, Ob, Ts, Ib (green leaf), Aa, Lc, Pp, Ba, Nc, Bc, or Am. Table 2 lists the amounts of flavonols in acidic methanolic extracts of plant leaves. Quercetin was the principle antioxidant constituent in Lc (12283.96 ± 171.17 μg/g DW), but no myricetin, morin, or kaempferol were detected in *Lycium chinense*. Leaves of Nc had the highest levels of myricetin at 1099.85 ± 37.03 μg/g DW. Morin was abundant in leaves of Al (20387.4 ± 1236.1 μg/g DW) compared to other plants. Interestingly, Bc contained only morin (315.79 ± 40.06 μg/g DW) while Ts had none. Kaempferol was only present in Gb (339.19 ± 301.01 μg/g DW, the highest level), Po, Ane, Ts, Pp, Hf (yellow), Nc, and Se (yellow) (36.36 ± 5.85 μg/g DW, the lowest level), but the others did not contain any kaempferol at all. Furthermore, no flavonols were found in Sn, Abe, Hf (green), Mc, and Am (green) plants. So and Ba contained quercetin and myricetin only; however, myricetin and morin were the principal flavonol constituents in Ac and Se (green). Thus, these indigenous Taiwanese vegetables display a wide variation in flavonol levels. Among the tested 27 vegetables, only six contained anthocyanidins, namely delphinidin (Del), cyanidin, malvidin (Mal), and pelargonidin (Pel) (Table A1). Both Cyan and Mal were abundant in Gb, Abe, Po, Ane, Ib-purple, and Hf (bright orange). Po had the highest levels of Cyan and Mal, with 12240.49 ± 284.99 μg/g DW and 105.29 ± 6.62 μg/g DW, respectively. Interestingly, Del was only detected in Abe, and none of the tested plants contained Pel.

**Table 1:** The content of various antioxidant substances in acid hydrolysates of 27 indigenous Taiwanese vegetables.

Sample *	Polyphenols mg GAE/g DW	Flavonoids mg QUE/g DW	Flavonols mg QUE/g DW	Anthocyanidins unit/g DW
Aa	4.26 ± 0.15 j	18.51 ± 3.50 g	3.46 ± 1.14 g	ND
Abe	3.24 ± 0.21 jk	14.31 ± 1.12 h	3.73 ± 0.45 g	5.27 ± 0.31 e
Ac	5.81 ± 0.37 i	40.44 ± 3.33 e	6.92 ± 2.73 ef	ND
Al	11.45 ± 0.54 fg	42.68 ± 4.62 de	10.84 ± 2.36 d	ND
Am (green)	6.41 ± 0.09 hi	45.40 ± 2.07 d	7.05 ± 2.41 e	ND
Am (red)	11.14 ± 0.05 fg	40.26 ± 1.49 e	5.87 ± 2.70 f	ND
Ane	12.94 ± 1.12 f	28.24 ± 4.17 f	15.26 ± 2.78 cd	4.85 ± 0.24 e
Ba	7.12 ± 1.40 h	42.71 ± 3.06 de	7.73 ± 2.19 e	ND
Bc	8.19 ± 0.16 h	39.31 ± 1.69 e	5.95 ± 1.93 f	ND
Gb	9.61 ± 0.34 gh	56.07 ± 5.03 c	7.59 ± 0.69 e	21.65 ± 0.02 d
Hf (bright orange)	2.90 ± 0.03 k	8.67 ± 0.36 h	3.47 ± 0.80 g	60.97 ± 6.79 c
Hf (green)	6.27 ± 0.33 hi	16.37 ± 1.23 gh	5.13 ± 3.38 f	3.48 ± 0.12 e
Ib (green)	18.17 ± 0.06 d	42.45 ± 2.38 de	7.80 ± 1.43 e	ND
Ib (purple)	22.80 ± 0.50 c	67.66 ± 9.76 b	23.82 ± 1.94 b	275.60 ± 26.21 b
Lc	9.24 ± 0.81 gh	46.80 ± 6.68 d	10.76 ± 2.70 d	ND
Li	15.15 ± 2.77 e	38.78 ± 3.25 e	15.13 ± 2.96 cd	ND
Mc	2.60 ± 0.81 k	19.28 ± 1.12 g	2.13 ± 0.14 h	0.31 ± 0.19 g
Nc	3.82 ± 1.02 jk	23.28 ± 0.54 f	6.71 ± 0.44 f	ND
Ob	23.78 ± 0.49 c	37.94 ± 4.93 e	13.01 ± 3.55 cd	ND
Po	40.80 ± 0.92 b	82.42 ± 7.34 a	28.43 ± 1.23 a	471.48 ± 36.40 a
Pp	10.02 ± 3.74 g	27.04 ± 0.10 f	3.15 ± 2.22 gh	ND
Se (green)	2.62 ± 0.52 k	4.27 ± 0.14 i	1.00 ± 0.22 i	1.42 ± 1.09 f
Se (yellow)	0.63 ± 0.18 l	1.87 ± 0.23 j	2.22 ± 0.42 h	1.12 ± 0.05 f
Sl	5.52 ± 0.42 i	38.56 ± 4.87 e	8.23 ± 2.24 de	ND
Sn	10.35 ± 0.28 g	45.40 ± 4.79 d	9.42 ± 0.62 de	ND
So	4.13 ± 0.69 j	ND	5.03 ± 1.10 fg	1.34 ± 0.20 f
Ts	81.86 ± 1.48 a	62.23 ± 4.62 bc	18.16 ± 0.87 c	ND

* Complete names of the tested samples are described in the *Plant materials*. All values are means ± SD (*n* = 3). Means within a column with different letters (a~l) are significantly different by LSD at *p* < 0.05. ND, not detected. GAE, gallic acid equivalent. QUE, quercetin equivalent. DW, dry weight.

**Table 2:** The content of flavonols in acidic methanolic hydrolysates of 27 indigenous Taiwanese vegetables.

Flavonols (μg/g DW)				
Sample *	Quercetin	Myricetin	Morin	Kaempferol
Aa	141.29 ± 25.24 i	ND	ND	ND
Abe	ND	ND	ND	ND
Ac	ND	781.28 ± 24.62 c	455.16 ± 70.32 h	ND
Al	172.85 ± 0.94 h	ND	20387.4 ± 1236.10 a	ND
Am (green)	ND	ND	ND	ND
Am (red)	51.41 ± 3.18 m	ND	1349.35 ± 5.36 f	ND
Ane	16.93 ± 1.44 o	48.21 ± 8.22 g	727.22 ± 57.17 g	209.61 ± 11.63 b
Ba	150.67 ± 3.62 i	184.81 ± 10.35 e	ND	ND
Bc	ND	ND	315.79 ± 40.06 i	ND
Gb	28.75 ± 3.52 w	ND	10945.10 ± 1125.64 c	339.19 ± 30.01 a
Hf (bright orange)	1140.12 ± 86.86 e	1037.64 ± 49.69 ab	3380.19 ± 337.42 e	142.89 ± 3.76 c
Hf (green)	ND	ND	ND	ND
Ib (green)	83.22 ± 2.74 k	ND	9376.21 ± 118.62 d	ND
Ib (purple)	852.63 ± 52.68 ef	ND	3266.11 ± 444.85 e	ND
Lc	12283.96 ± 171.17 a	ND	ND	ND
Li	9397.28 ± 56.78 b	13.37 ± 1.30 h	16811.42 ± 727.43 b	ND
Mc	ND	ND	ND	ND
Nc	116.53 ± 26.66 jk	1099.85 ± 37.03 a	3869.54 ± 213.27 e	107.41 ± 5.35 d
Ob	2764.84 ± 228.19 d	ND	4091.51 ± 382.99 e	ND
Po	7773.14 ± 353.67 c	53.08 ± 7.71 fg	16766.97 ± 541.42 b	70.88 ± 4.20 e
Pp	476.41 ± 61.30 g	290.34 ± 27.65 d	203.72 ± 23.80 j	42.26 ± 4.95 f
Se (green)	ND	756.13 ± 49.99 c	194.99 ± 6.89 j	ND
Se (yellow)	64.86 ± 2.86 l	1010.54 ± 31.05 b	404.38 ± 82.33 hi	36.36 ± 5.85 f
Sl	130.64 ± 13.29 ij	ND	8984.97 ± 502.52 d	ND
Sn	ND	ND	ND	ND
So	0.42 ± 0.14 p	3.41 ± 1.14 i	ND	ND
Ts	54.78 ± 5.06 m	62.80 ± 2.44 f	ND	226.62 ± 35.72 b

All values are means ± SD (*n* = 3). Means within a column with different letters (a~p) are significantly different by LSD at *p* < 0.05. ND, not detected.

### 3.2. Antioxidant Activity of DPPH Radical Scavenging, TEAC Values, and ORAC Values with Methanol and Ethanol Extracts

Table 3 demonstrates that plant methanolic extracts have significant differences in their ability to scavenge DPPH, exhibiting a wide range of scavenging values from 1.75 ± 0.26 μg/mL (Ts plant) to 1801.56 ± 3.16 μg/mL (Se green leaf plant). In addition, plant extracts from all species also show an antioxidant capacity for scavenging the ABTS radical cation (the antioxidant activity in methanolic extracts and acidic ethanolic extracts of leaf tissues is expressed in Trolox equivalent antioxidant capacity). Ts had significantly higher values of TEAC-methanolic (1218.77 ± 115.51 Trolox μmol/g DW) and TEAC-ethanolic (128.99 ± 5.39 Trolox μmol/g DW) than other species. All methanolic extracts had higher TEAC values than ethanolic extracts in all samples, indicating that methanolic extracts are more effective than ethanolic extracts in scavenging ROS. Furthermore, all extracts exhibited distinct antioxidant activities with oxygen radical absorption capacity (ORAC-hydrophilic) values from 2701.16 ± 95.68 μmol Trolox/g DW (Po) to 48.86 ± 2.90 μmol Trolox/g DW (So), while ORAC-lipophilic values ranged from 359.91 ± 20.96 μmol Trolox/g DW (So) to 33.54 ± 0.22 μmol Trolox/g DW (Hf-bright orange) (Table 4). A high ORAC value means that a sample is high in antioxidants. Most of the selected indigenous vegetables had higher ORAC-hydrophilic values than ORAC-lipophilic values, indicating that hydrophilic extracts are more effective than lipophilic extracts in scavenging ROS, and that these plants are high in antioxidants.

**Table 3:** The IC_50_ of DPPH scavenging activity and TEAC values in acid methanolic hydrolysates and ethanolic extracts of 27 indigenous Taiwanese vegetables.

Sample	DPPH scavenge IC_50_ (μg/mL)	TEAC-methanolic (μmol Trolox/g DW)	TEAC-ethanolic (μmol Trolox/g DW)
Aa	616.13 ± 12.21 k	32.43 ± 2.65 h	1.73 ± 0.82 kl
Abe	226.57 ± 0.69 p	38.39 ± 4.43 gh	3.73 ± 0.58 j
Ac	1173.32 ± 7.56 d	36.22 ± 3.54 gh	21.04 ± 2.75 e
Al	76.99 ± 0.13 t	89.00 ± 13.27 d	0.48 ± 0.13 mn
Am (green)	1505.11 ± 1.52 b	15.77 ± 0.54 k	0.61 ± 0.08 m
Am (red)	1027.74 ± 4.08 f	30.33 ± 2.04 hi	1.19 ± 0.14 lm
Ane	130.72 ± 3.24 s	80.52 ± 1.35 de	13.45 ± 0.76 f
Ba	427.78 ± 0.48 o	45.96 ± 0.69 ef	1.57 ± 0.41 l
Bc	492.37 ± 1.85 m	40.85 ± 0.53 g	1.90 ± 0.50 kl
Gb	893.34 ± 15.37 g	36.34 ± 1.25 gh	3.99 ± 0.94 j
Hf (bright orange)	22.80 ± 0.14 v	48.81 ± 14.13 e	8.14 ± 0.03 h
Hf (green)	206.25 ± 0.52 q	39.75 ± 2.76 gh	7.15 ± 0.90 hi
Ib (green)	74.67 ± 0.06 t	92.76 ± 6.83 d	10.66 ± 0.93 g
Ib (purple)	803.13 ± 0.38 i	162.66 ± 11.73 c	25.58 ± 0.66 d
Lc	547.74 ± 0.43 l	94.68 ± 6.24 d	9.10 ± 0.11 gh
Li	165.39 ± 0.26 r	79.37 ± 12.60 de	1.99 ± 0.82 kl
Mc	1119.78 ± 0.26 e	27.76 ± 1.92 i	0.25 ± 0.05 n
Nc	34.01 ± 0.11 u	47.80 ± 2.84 ef	6.12 ± 0.25 i
Ob	22.21 ± 0.07 v	114.56 ± 7.68 d	34.10 ± 1.24 c
Po	17.27 ± 0.25 w	339.44 ± 6.95 b	71.37 ± 2.97 b
Pp	688.52 ± 2.06 j	31.56 ± 3.54 hi	1.53 ± 0.01 l
Se (green)	1801.56 ± 3.16 a	21.70 ± 2.91 j	2.32 ± 0.29 k
Se (yellow)	1503.96 ± 2.57 c	18.09 ± 0.80 j	1.28 ± 0.24 n
Sl	811.70 ± 4.27 h	43.08 ± 0.90 ef	21.26 ± 0.21 e
Sn	471.5 ± 0.66 n	40.26 ± 1.92 g	1.19 ± 0.11 lm
So	867.38 ± 14.95 gh	3.33 ± 0.07 l	0.56 ± 0.04 m
Ts	1.75 ± 0.26 x	1218.77 ± 115.51 a	128.99 ± 5.39 a

All values are meanis ± SD (*n* = 3). Means within a column with different letters (a–x) are significantly different by LSD at *p* < 0.05. ND, not detected. DPPH, 1,1-Diphenyl-2-picryl-hydrazyl. TEAC, trolox equivalent antioxidant capacity.

**Table 4:** ORAC values in acid methanolic hydrolysates of 27 indigenous Taiwanese vegetables.

Sample	Hydrophilic ORAC (μmol Trolox/g DW)	Lipophilic ORAC (μmol Trolox/g DW)
Aa	181.68 ± 28.75 i	148.60 ± 3.88 d
Abe	187.58 ± 28.82 i	146.60 ± 10.70 d
Ac	202.59 ± 9.62 i	157.75 ± 8.47 d
Al	680.86 ± 47.02 d	145.02 ± 5.60 d
Am (green)	59.24 ± 1.67 l	135.34 ± 7.72 e
Am (red)	79.88 ± 18.68 k	71.40 ± 0.07 h
Ane	457.62 ± 47.83 ef	164.92 ± 16.07 c
Ba	513.79 ± 8.75 e	148.13 ± 3.73 d
Bc	243.50 ± 17.66 h	183.47 ± 16.45 bc
Gb	117.69 ± 12.71 jk	159.17 ± 7.37 cd
Hf (bright orange)	406.40 ± 17.48 f	33.54 ± 0.22 j
Hf (green)	129.46 ± 14.03 j	183.60 ± 20.67 bc
Ib (green)	786.26 ± 15.38 c	143.65 ± 13.32 de
Ib (purple)	1174.98 ± 135.08 b	92.43 ± 2.15 f
Lc	339.37 ± 27.23 g	149.27 ± 7.04 d
Li	662.25 ± 9.39 d	160.44 ± 10.16 cd
Mc	85.38 ± 19.10 k	148.02 ± 12.82 d
Nc	129.32 ± 8.00 j	175.86 ± 1.09 bc
Ob	692.18 ± 16.45 d	144.40 ± 15.50 de
Po	2701.16 ± 95.68 a	70.07 ± 1.80 h
Pp	191.55 ± 27.55 i	137.94 ± 4.19 e
Se (green)	49.95 ± 7.94 l	38.97 ± 0.27 i
Se (yellow)	120.29 ± 6.91 j	34.30 ± 0.82 j
Sl	141.56 ± 17.64 j	196.67 ± 33.91 b
Sn	103.23 ± 26.66 jk	74.63 ± 1.62 g
So	48.86 ± 2.90 l	359.91 ± 20.96 a
Ts	1284.04 ± 123.99 b	147.10 ± 5.98 d

All values are means ± SD (*n* = 3). Means within a column with different letters (a–k) are significantly different by LSD at *p* < 0.05. ORAC, oxygen radical absorption capacity.

### 3.3. Correlations between Antioxidant Activity and Antioxidant Substances in Indigenous Taiwanese Vegetables

The IC_50_ of DPPH was significantly and negative correlated with polyphenols (*r* = −0.43, *p* = 0.0215) and morin (*r* = −0.39, *p* = 0.034) (Table 5). TEAC-methanolic values were positively correlated with polyphenols (*r* = 0.95, *p* < 0.0001), kaempferol (*r* = 0.42, *p* = 0.0228), and ORAC-hydrophilic values (*r* = 0.56, *p* = 0.0017) (Table 5 and Table 6). Positive correlations were also found among TEAC-ethanolic values and flavonoids (*r* = 0.55, *p* = 0.0022), flavonols (*r* = 0.65, *p* = 0.0002), and ORAC-hydrophilic values (*r* = 0.71, *p* < 0.001). However, no significant correlations were observed among ORAC-lipophilic values and other antioxidant activities and antioxidant substances (data not shown). Table 6 demonstrates that ORAC-hydrophilic values were most positively or negatively (DPPH radicals scavenging IC_50_ only) correlated with all antioxidant activities and antioxidant substances except myricetin and kaempferol. Thus, different antioxidant substances displayed varying levels of antioxidant activity.

**Table 5:** Correlations between IC_50_ of DPPH radical scavenging and TEAC values *versus* antioxidant substances and activities of 27 indigenous Taiwanese vegetables.

Parameter	DPPH scavenge IC_50 _(μg/mL)	TEAC-methanolic (Trolox μmol/g DW)	TEAC-ethanolic (Trolox μmol/g DW)
Polyphenols	*r* = −0.43 *p* = 0.0215	*r* = 0.95 *p* < 0.0001	-
Anthocyanidins	NS	NS	-
Flavonoids	-	-	*r* = 0.55 *p* = 0.0022
Flavonols	-	-	*r* = 0.65 *p* = 0.0002
Quercetin	NS	NS	NS
Myricetin	*r* = −0.51 *p* = 0.0048	NS	NS
Morin	*r* = −0.39 *p* = 0.034	NS	NS
Kaempferol	NS	*r* = 0.42 *p* = 0.0228	NS

NS, no significant correlation. -, not analyzed.

**Table 6:** Correlation among ORAC values *versus* antioxidant substances and activities of 27 indigenous Taiwanese vegetables.

Parameter	Hydrophilic ORAC (μmol Trolox/g DW)
Polyphenols	*r* = 0.72 *p*<0.0001
Anthocyanidins	*r* = 0.84 *p* < 0.0001
Flavonoids	*r* = 0.69 *p* < 0.0001
Flavonols	*r* = 0.87 *p*<0.0001
Quercetin	r = 0.54 *p* = 0.0208
Myricetin	NS
Morin	*r* = 0.53 *p* = 0.0035
Kaempferol	NS
TEAC-methanolic	*r* = 0.56 *p* = 0.0017
TEAC-ethanolic	*r* = 0.71 *p* < 0.0001
DPPH radicals scavenging IC_50_	*r* = −0.47 *p* = 0.011

NS, no significant correlation.

## 4. Discussion—Antioxidant Composition and Activity

Po leaves contained relatively more polyphenols, flavonoids, flavonols, and anthocyanidins compared to other tested plants (Table 1). Ts had a similar trend except for anthocyanidins. Lc, Li, and Po contained abundant quercetin, while Al and Gb were rich in morin and kaempferol, respectively (Table 2). Additionally, Nc and Se (yellow) plants had significantly higher levels of myricetin than other tested samples. Ts and Po displayed higher TEAC values and lower DPPH radical scavenging IC_50_ values than the other plants (Table 3). The TEAC-methanolic values reported here are similar those of Yang *et al.* [28] regarding Ob, Gb, and Ib (green), except that the latter had lower TEAC values. Proteggente *et al.* [29] reported that the TEAC values of their tested fruits and vegetables were correlated with values measured by other widely used methods, including ORAC, total radical antioxidant potential (TRAP), and total phenolics. In our study, TEAC values were correlated with polyphenols (*r* = 0.95), flavonoids (*r* = 0.55), flavonols (*r* = 0.65), and kaempferol (*r* = 0.42) (Table 6). Moreover, ORAC-hydrophilic values were correlated with TEAC values in methanolic and ethanolic extracts (*r* = 0.56 and 0.71, respectively) (Table 6). Antioxidant activities are known to increase proportionally to polyphenol content, mainly due to their redox properties [30,31]. The ability to act as an antioxidant depends on the chemical structure and ability to donate/accept electrons, thus delocalizing an unpaired electron within an aromatic structure [18]. Furthermore, phenolic compounds are known to be radical scavengers or radical-chain breakers, and they strongly eliminate oxidative free radicals. Quercetin and morin were the principal flavonol constituents in leaves of Lc and Al, respectively (Table 2), and may account for the high ORAC-hydrophilic activity measured in the present study (Table 4). The ORAC-hydrophilic value of Ob was close to the Ob value in the USDA ORAC databank [32], while Po, Ts, and Ib (both purple and green) had higher ORAC-hydrophilic values. Meanwhile, Li and Al contained the same level of ORAC-hydrophilic values as those of the USDA. ORAC levels in common vegetable samples (such as white cabbage, carrot, snap bean, cauliflower, white onion, purple onion, broccoli, tomato, beet, pea, spinach, red pepper, and green pepper) ranged from 19 µmol Trolox/g DW in the pea to 154 µmol Trolox/g DW in green peppers [27], which were much lower than any of the 27 vegetables that we tested (Table 4). ORAC reflects peroxyl radical scavenging activity and represents a hydrogen atom transfer (HAT) reaction mechanism [27,33]. The Folin-Ciocalteu method is an electron transfer (ET)-based assay that estimates reducing capacity, and has usually been expressed as a phenolic content. The TEAC assay represents a second ET-based method [33] that uses exogenous ABTS radicals, whereas the ORAC assay uses peroxyl radicals. Because peroxyl radicals are the most common radicals found in the human body, ORAC measurements should be more biologically relevant [33]. Biologically relevant reactive oxygen species (ROS) include O_2_‒•, HO•, ONOO‒, and singlet oxygen. Furthermore, ORAC assay measures only the antioxidant capacity against peroxyl and hydroxyl radicals but not all reactive oxygen species [34]. The ORAC assay measures antioxidants using AAPH (a ROO• generator) including ascorbic acid, α-tocopherol, β-carotene, glutathione, bilirubin, uric acid, and melatonin. Cu^2+^-H_2_O_2_ (an OH• generator) is used for measuring compounds, such as glucose, mannitol, uric acid, and transition metal chelators in the ORAC assay [34,35]. TEAC method is based on the oxidation of ABTS in the presence of H_2_O_2_ and metmyoglobin, and only measures hydrophilic antioxidants. Preparation of ABTS radical by filtering ABTS solution through manganese dioxide powder allows TEAC assay to measure lipophilic antioxidants. In ethanol solvent, TEAC method enables the measurement of both hydrophilic and lipophilic antioxidants [34,36]. However, the ABTS radical used in TEAC assays is not found in biological systems [34,37]. The DPPH assay is technically simple and rapid. DPPH can only be dissolved in organic media but not in aqueous media, which is an important limitation when interpreting the role of hydrophilic antioxidants [34]. As different ROSs have different reaction mechanisms, at least two methods are recommended for evaluating antioxidant activity [27,33,34]. The total antioxidant capacity values should include those assays applicable to both lipophilic and hydrophilic antioxidants and relate to the similarity and differences of both HAT and ET [33,34]. The antioxidant action of anthocyanidins serve as quenching ROS, while flavonoids serve as chain-breaking antioxidants which scavenge ROS [34,38].

Previously, we demonstrated that the content of polyphenols, flavonoids, and anthocyanidins in Taiwan’s indigenous purple-leaved vegetables were much higher than in green vegetable controls [12]. In the present study, the polyphenol and anthocyanidin levels of these 27 plants (Table 1) were much lower than those of our previous study, where Ib-purple (33.4 ± 0.5 mg gallic acid/g DW) and Po (1444.1 ± 42.2 unit/g DW) had higher levels [12]. However, in our current study, the polyphenol content of Ts and Po was much higher than in indigenous purple-leaved vegetables [12]. Yang *et al.* [39] reported the flavonoid content of 91 species, including popular and lesser-known plants consumed as vegetables and spices in tropical and sub-tropical areas of Asia. Leaves of Indian mulberry, ashitaba, Chinese cedar, Vietnamese coriander, and moringa had the highest levels of total flavonoids at 254, 155, 144, and 129 mg/100 g FW, respectively. These plants were rich in quercetin and kaempferol, and more than 50% of these plants contained either or both quercetin and kaempferol, while quercetin was the major type of flavonoid. Furthermore, quercetin was found in the shoots of Am, Lc, Sn, Ib-green, and Ib-purple at 2.63, 0.61, 0.46, 0 and 3.25 mg/g DW, respectively, but less or even no kaempferol was detected in these plants [39]. The quercetin levels of the 27 indigenous vegetables were much higher than reported by Yang *et al.* [39], especially for Ob, Lc, Li and Po at 2.76 mg/g DW, 12.28 mg/g DW, 9.40 mg/g DW and 7.77 mg/g DW, respectively, but these vegetables contained no kaempferol at all (Table 2). The highest total flavonoid values reported in the USDA database [32] for raw vegetables are capers (*Capparis*, 28.99 mg/g DW), parsley (11.85 mg/g DW), lovage (*Levisticum officinale*, 17.7 mg/g DW), dill weed (*Anethum graveolens*, 6.12 mg/g DW), and dock (*Rumex* spp., 5.10 mg/g DW). The USDA flavonoid database (release 3.1) [40] lists raw sweet potato leaves as being abundant in quercetin (1.30 mg/g DW), myricetin (0.34 mg/g DW), kaempferol (0.16 mg/g DW), and some flavones (luteolin and apigenin), while raw perilla leaves are rich in quercetin (0.03 mg/g DW), myricetin (0.02 mg/g DW), and some flavones (luteolin and apigenin). In our study, Po had much higher quercetin (7.77 mg/g DW) and myricetin (0.053 mg/g DW) compared to USDA perilla leaf data (Table 2). In addition, Ib-purple and Ib-green plants contained quercetin and morin only, and at higher levels than the USDA list indicates. In our previous study, Ib (purple) and Po were also abundant in quercetin at 2.44 and 2.64 mg/g DW, respectively. Meanwhile, Ib-purple and Po were rich in cyanidin (0.51 and 0.31 mg/g DW, respectively) and malvidin (0.4 and 0.33 mg/g DW, respectively) [12]. In the present study, more than half of the 27 selected plants contained quercetin, morin, and myricetin. Quercetin and kaempferol were found to be the major and minor types of flavonols, respectively (Table 2). Po also contained abundant in cyanidin (12.24 mg/g DW, Table A1), which was much higher than reported in our previous study. Moreover, both Ane (dark green leaf) and Hf (bright orange leaf) also contained anthocyanidin (Table 2) and one of its constituents, cyanidin (Table A1). The data from our study, therefore, provide additional information for flavonol and anthocyanidin databases and contribute to studies on the health benefits of flavonol and anthocyanidin intake, especially for populations consuming tropical and under-utilized vegetables. 

## 5. Conclusions

The 27 tested indigenous Taiwanese vegetables showed high levels of polyphenols, flavonoids, and flavonols that correlated well with antioxidant activity. Po, Ts, and Ib (purple leaf) exhibited higher antioxidant activity that may be related to their higher cyanidin, quercetin, and polyphenol levels. 

## Appendix

**Table A1:** The content of anthocyanidins in acidic methanolic hydrolysates of indigenous Taiwanese vegetables.

Anthocyanidins (μg/g DW)				
Sample	Delphinidin	Cyanidin	Pelargonidin	Malvidin
Aa	ND	ND	ND	ND
Abe	24.34 ± 8.47	114.10 ± 0.79 e	ND	14.91 ± 0.21 e
Ac	ND	ND	ND	ND
Al	ND	ND	ND	ND
Am (green)	ND	ND	ND	ND
Am (red)	ND	ND	ND	ND
Ane	ND	1119.18 ± 86.91 c	ND	ND
Ba	ND	ND	ND	ND
Bc	ND	ND	ND	ND
Gb	ND	691.85 ± 39.21 d	ND	53.61 ± 0.47 b
Hf (bright orange)	ND	627.02 ± 47.60 d	ND	17.88 ± 0.10 d
Hf (green)	ND	ND	ND	ND
Ib (green)	ND	ND	ND	ND
Ib (purple)	ND	1437.32 ± 33.87 b	ND	30.37 ± 0.27 c
Lc	ND	ND	ND	ND
Li	ND	ND	ND	ND
Mc	ND	ND	ND	ND
Nc	ND	ND	ND	ND
Ob	ND	ND	ND	ND
Po	ND	12240.49 ± 284.99 a	ND	105.29 ± 6.62 a
Pp	ND	ND	ND	ND
Se (green)	ND	ND	ND	ND
Se (yellow)	ND	ND	ND	ND
Sl	ND	ND	ND	ND
Sn	ND	ND	ND	ND
So	ND	ND	ND	ND
Ts	ND	ND	ND	ND

All values are means ± SD (*n* = 3). Means within a column with different letters (a~e) are significantly different by LSD at *p* < 0.05. ND: not detected. DW: dry weight.
